# The impact of home, work, and church environments on fat intake over time among rural residents: a longitudinal observational study

**DOI:** 10.1186/s12889-016-2764-z

**Published:** 2016-01-29

**Authors:** Regine Haardörfer, Iris Alcantara, Ann Addison, Karen Glanz, Michelle C. Kegler

**Affiliations:** 1 Department of Behavioral Sciences and Health Education, Rollins School of Public Health, Emory University, 1518 Clifton Road, Atlanta, GA 30322 USA; 2 Emory Prevention Research Center, Atlanta, GA USA; 3 Primary Care of Southwest Georgia, Inc, Blakely, GA USA; 4 School of Nursing, University of Pennsylvania, Philadelphia, PA USA

**Keywords:** Fat intake, Socio-ecological model, Longitudinal, Rural

## Abstract

**Background:**

Dietary behaviors are influenced by many individual and environmental factors. This study explores how dietary fat intake in high-risk midlife adults living in the rural south is influenced by three behavior settings, i.e. in the home, at work, and at church.

**Methods:**

Self-report data were collected from rural African American or Caucasian adults age 40–70 at three time points at baseline, 6, and 12 months post baseline. Multilevel analyses investigated the impact of determinants of fat intake over time.

**Results:**

Home and work environments varied significantly over time in regard to healthy eating while church environments remained stable. Age, gender, and self-efficacy for healthy eating were individual factors associated with fat intake. In the home, presence of more high fat items, a time-varying variable, was significant. In the work environment, having access to healthy foods as well as healthy eating programs has positive impact as did hearing healthy eating messages and availability of healthy foods at church.

**Conclusions:**

Understanding stability and variability of dietary fat intake from a social ecologic perspective will aid in identifying targets of change for intervention. Understanding which components of key behavior settings are dynamic and which are relatively stable will help to disentangle the complexity of multi-level determinants of dietary behavior.

## Background

Ecologic models are widely acknowledged as powerful tools for understanding behaviors that contribute to obesity [1, 2]. Dietary behaviors, such as fat intake, can be explained by a complex interplay of biological, psychological, cultural and social factors, combined with behavior settings, organizational and community contexts, and public policies [1, 3, 4]. This complexity is difficult to study cross-sectionally and even more so longitudinally [5, 6]. Research that examines associations between environments and behavior is often cross-sectional, as is most research based on a social ecologic approach [7–10]. Moreover, the few studies that examine these associations longitudinally rarely examine multiple behavior settings or microenvironments, such as homes and workplaces, simultaneously [11–13].

**Fig. 1:**
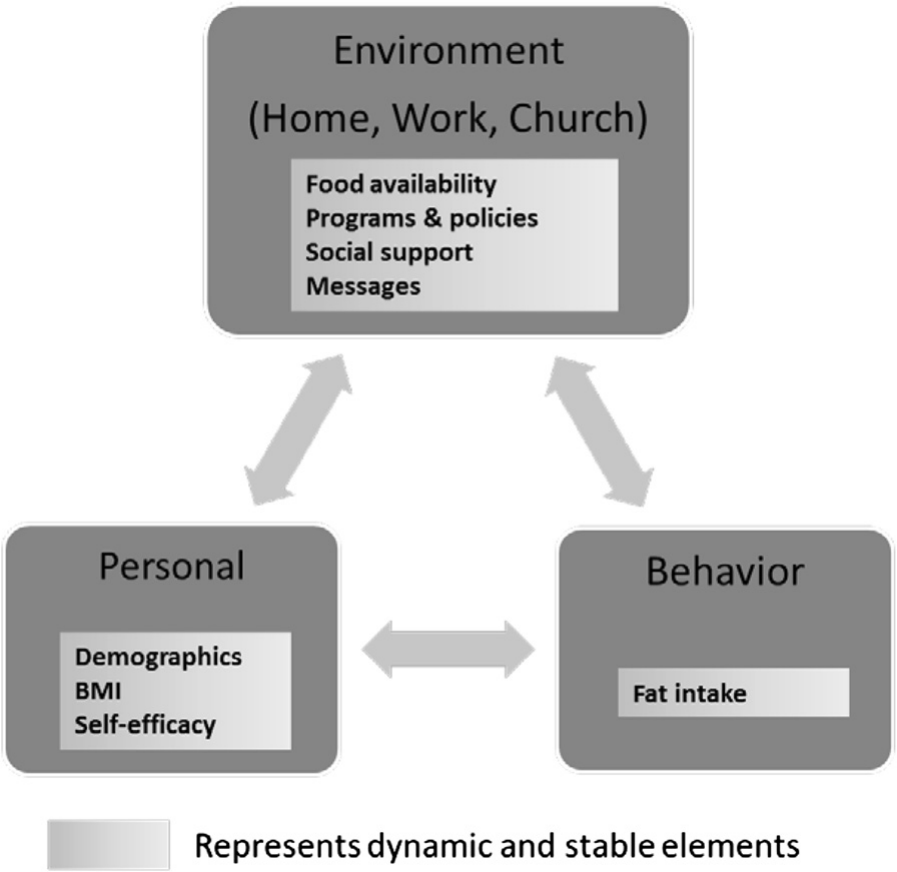
Conceptual Model for Understanding Fat Intake from a Social Cognitive Perspective

The current study explores how dietary fat intake in high-risk midlife adults living in the rural south is influenced by three behavior settings over time. We operationalized the Social Ecological Model for healthy eating by focusing on physical and social aspects of three priority environments in combination with individual factors [14]. Figure 1 presents this conceptual model and includes individual and environmental factors, along with social and physical aspects of home, workplace and church environments. Of particular note, all variables are considered dynamic as they may vary over time. This conceptualization is consistent with the notion of reciprocal determinism from social cognitive theory [15] and the bidirectional and dynamic relationships implied in ecologic models of behavior [14, 16–18].

Controlling dietary fat intake, especially saturated fats, is recommended by the Institute of Medicine and the World Health Organization, among others [19]. High dietary fat intake has been linked to increased risk of some cancers and is a major risk factor for cardiovascular disease [20]. Additionally, the high energy density of fat facilitates excessive caloric intake which can lead to weight gain and associated health risks, including cancer, cardiovascular disease and diabetes [21].

The home environment is an important behavior setting for affecting fat intake because a significant proportion of calories are still consumed or prepared at home [22]. Household food availability is related to food consumption [4, 23, 24]. Patterson and associates [25] found that living in a household with fewer high fat foods was associated with lower fat intake. In addition, social support from family members also predicts dietary behavior [26, 27].

Workplaces are another behavior setting with significant implications for dietary behavior. The majority of adults in the U.S. spend over 8 h a day at work [28, 29]. Access to healthy foods at work has an impact on what workers consume [30]. Booth and associates [3] identify a long list of workplace influences on eating behavior, including kitchen facilities, cafeterias, vending machines and eating policies. Numerous worksite programs to promote healthy eating have shown positive effects on dietary intake [11, 31].

The church is a third behavior setting with the potential to shape eating behavior [32, 33]. It may be particularly relevant in the rural south. Approximately 60 % of churches in the U.S. are in or near small towns [34]. Food plays an important role in many church related activities [35]. Churches are also places of social support for healthy eating [33, 35] and increasingly common settings for nutrition programs [36].

Relatively little research exists on the stability of the physical and social aspects of these eating behavior settings. Does family support for healthy eating remain stable over time? If it changes, what impact does it have on healthy eating behavior? Do household food inventories vary significantly and how does that impact food intake? To what extent do work or church-based food policies remain stable? Do programs available through church or the workplace remain in place over time? Qualitative studies in South Georgia suggest that in rural areas, programs in small worksites and churches tend to be short-lived [35, 37, 38]. Research on household food inventories suggests that food availability may change significantly week to week [39]. In addition, self-efficacy for healthy eating has also been linked to lower fat intake for women [40].

Understanding stability and variability of dietary fat intake from a social ecologic perspective will aid in identifying targets of change for intervention. Furthermore, understanding which components of key behavior settings are dynamic and which are relatively stable will help the field begin to disentangle the complexity of multi-level determinants of dietary behavior. Few studies have looked simultaneously at the impact of the home, work, and church environment on fat intake behavior across time. This longitudinal study used community-based participatory research to investigate individual and contextual determinants of fat intake in rural adults with or at high risk for cardiovascular disease. The following research questions guided our analyses:

1. How stable are individual determinants as well as environmental determinants in the home, at work, and at church?
2. How do stable and dynamic individual determinants of fat intake, in combination with environmental determinants in home, work, and church, impact fat intake?

## Methods

### Study description

Healthy Rural Communities 2 is a community based participatory study conducted in four rural counties without urbanized areas (US Census 2000) (Brooks, Sumter, Worth, and Decatur) in southwest Georgia [41]. This study was designed to examine determinants of healthy eating and physical activity explored in a previous qualitative study by the Emory Prevention Research Center [36, 38, 42]. Community Advisory Board (CAB) members collaborated in all research activities to varying degrees, from developing research questions to survey development to interpretation of results. Data were collected at baseline (*n* = 527) and, for a subset (*n* = 245) of participants who were at high risk for cardiovascular disease, 6 and 12 months later between 2006 and 2008. Baseline data were collected in-person after consent was obtained using a self-administered instrument. Participants in the study were recruited from various community settings, including local businesses, restaurants, churches, libraries, health departments, and civic organizations. Six and 12 month data were collected by telephone. Waist circumference at baseline was measured by study staff. Participants were each given a $20 gift card upon completion of each data collection point. The study was approved by the Emory University Institutional Review Board.

Participants in the study were African American or Caucasian adults age 40–70; lived with at least one other person; and were residents of their respective counties for at least 5 years. Only one person per household was enrolled in the study, and individuals who had cancer or received cancer treatment in the past 2 years were excluded from the study.

For the 6 and 12 month follow up, 333 participants with or at high risk for cardiovascular disease were identified and of those 245 were reached for data collection at both follow up time points. All smokers were included. Additionally, for men, this sample consisted of individuals who had a waist circumference greater than 40 in. or weighed more than 190 lb or had a BMI of at least 30; and had at least one chronic disease (high blood pressure, coronary heart disease, stroke, diabetes, high cholesterol, or lung disease such as emphysema or chronic obstructive lung disease). For women, similar criteria were used except for waist circumference, the inclusion criterion was at least 35 in. and for weight, the inclusion criterion was greater than 160 lb. Rates of smokers, and participants with chronic diseases are presented in Table 1. Analysis reported here includes only individuals with data at all three time points (*n* = 245, 73.6 %).

**Table 1:** Baseline demographics (*N* = 245)

	Number	Percent
Gender		
Male	105	42.9
Female	140	57.1
Age		
< 50	97	39.6
50–60	101	41.2
≥ 60	47	19.2
Race		
White	130	53.1
Black	115	46.9
Marital status		
Married/living w/ partner	155	64.6
Separated/divorced	48	20.0
Widowed	13	5.4
Never married	24	10.0
Education		
< HS graduate	28	11.5
HS/GED	72	29.5
Some college	81	33.2
College graduate	44	18.0
Post grad/ prof. degree	19	7.8
Weight status at baseline		
Under/normal weight	31	12.9
Overweight	83	34.4
Obese	127	52.7
Smoking status		
Daily smoker	75	30.9
Non-daily smoker	17	7.0
Non-smoker	151	62.1
Chronic disease (% yes)		
High blood pressure	145	59.4
Coronary heart disease	18	7.5
Stroke	6	2.5
Diabetes	63	26.1
High cholesterol	104	43.7

#### Description of measures/variables

**Fig. 2:**
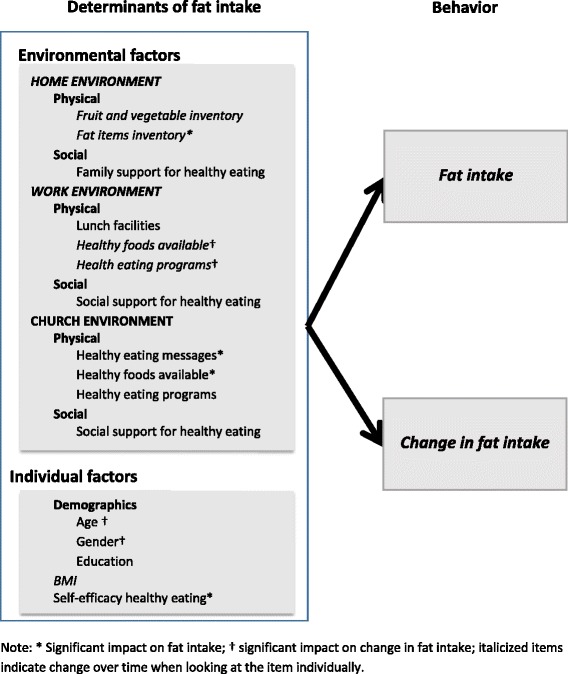
Overview of variables included in the growth model and results from all analyses

The conceptual framework presented in Fig. 2 shows the relationship between personal and environmental variables and behavior.

Fat intake was assessed using six items from the NCI fat screener developed by Thompson and colleagues [43]. Items included regular fat sausage or bacon; regular fat cheese or cheese spread; French fries or hash browns; regular fat mayonnaise; regular fat salad dressings; and margarine, butter, or oil. Response options ranged from never or less than once per month to five or more times per week.

BMI was determined using self-reported height and weight data using questions from the 2005 Behavioral Risk Factor Surveillance System [44].

Self-efficacy for healthy eating (α = 0.78) was assessed using six items adapted from the Eating Habits Confidence Survey developed by Sallis and colleagues [45]. Participants were asked, from a scale of 1 = I definitely can’t to 5 = I definitely can, how confident they were about eating low fat and low salt foods, eating smaller portions at dinner, and eating less red meat.

Participants also reported age, gender and level of education. Education was dichotomized into two categories; high school diploma/GED or less and more education.

##### Environment: Home

Home Food Inventory–Fruits and vegetables in the home were inventoried using seven items [25]. The inventory included bananas, apples, oranges, peaches, watermelon, carrots, and tomatoes. High fat foods were inventoried using three items: regular bacon or sausage, regular whole milk, and regular hot dogs [25]. Questions were asked in yes/no format. The inventories were reduced in collaboration with the CAB. Summed scores were calculated for fruits and vegetables and regular fat times.

Family Support for Healthy Eating–Family support for healthy eating was assessed using seven items adapted from the Social Support and Eating Habits Survey developed by Sallis and colleagues [46]. Participants reported how often their family or anyone living in their household discussed healthy and unhealthy dietary behaviors. Negative items were reverse scored before calculating the mean across items.

##### Environment: Church

Social Support for healthy eating at church–Two items adapted from Sallis and associates [47] were used to assess social support for healthy eating at church. Similar to previous social support questions, participants were asked, in the past 6 months, how often anyone from their church discussed their eating habit changes with them and how often someone at their church offered them foods they are trying not to eat. Answer choices ranged from 1 = never or rarely to 4 = almost always.

In addition to social support for healthy eating at church, we also assessed health messages at church, including messages about eating healthy in sermons [48] and health messages included on church bulletins or newsletters [49]. Additionally, questions about the availability of baked, broiled, or grilled chicken or fish, and friend chicken or fish, and one question about the availability of nutrition programs at church were included [49]. For the multivariable analyses, we coded those who reported any messages versus those who reported none. Access to food programs at church was treated the same. A food score was calculated by adding 1 for availability of each healthy food and 1 for the absence of each unhealthy food. The score was dichotomized into 0 (food score >0) and 1 (food score = 0).

##### Environment: Work

Work Environment–Facilities for food storage and preparation were assessed using items adapted from the CHEW Tool (Checklist for Health Promotion Environments at Worksites) [50]. We asked about microwaves, refrigerators, ovens or toasters, and break rooms in the workplace. The availability of healthy food options at cafeterias, snack bars, or food service, and the availability of healthy food options at vending machines. Additionally, we assessed the availability of nutrition and weight loss programs at the workplace [50]. For the multivariable analyses, we coded the presence of any facilities for lunch as 1 and otherwise as 0. Presence of healthy foods and programs for healthy eating was coded the same way, 1 for any and 0 otherwise.

Social Support for healthy eating at work–Questions adapted from Sallis and associates [46] were used to assess social support for healthy eating from coworkers. Participants were asked, in the past 6 month, how often their coworkers discussed their eating habit changes and encouraged them to eat healthy foods.

All church and work variables were dichotomized to include those participants who did not attend church regularly or did not work outside the home. All items were coded as providing no support if no or little support were indicated and as providing support when more support was reported. Data were collected at three time points for items where short term change was expected such as the home environment and social support in all environments. For items assessing the more stable physical work and church environments, data were only included at baseline and 12 months follow-up.

### Statistical analysis

Data were analyzed using SAS 9.3. Missing data was a minor problem (See Table 1) and hence listwise deletion was used in the multivariable analyses.

The main analysis approach for this paper was hierarchical linear growth modeling [51, 52] of fat intake over time. First, descriptive analyses were conducted for the outcome variable fat intake and all other variables. The main outcome variable, percentage of calories from fat, was distributed normally. We then assessed which factors were time invariant and which were time-varying as this determined how they were modeled in the subsequent hierarchical linear growth analysis.

For those variables collected only at baseline and 12 month, dependent t-tests (continuous variables) and McNemar’s test (categorical variables) were conducted to assess if the change over time was significant. Those 2 wave variables where change was not significant were classified as time-invariant and baseline values were used. Otherwise, a change score was calculated and modeled as an interaction with time. For variables that were collected at all three time points, unconditional growth model analyses were conducted to assess significance of change over time. Variables were modeled as time-varying and time-invariant (using baseline values) in subsequent analyses.

The multilevel analysis building involved a succession of four nested models and all included dummy variables for county as control variables [52]. The intercept and the time slope were modeled as random effects, allowing participants to vary randomly in their baseline fat intake and their change in fat intake. The full model tested was:

$$ (L1)\  FatIntak{e}_{ti}={\pi}_{0i}+{\pi}_{1i}Tim{e}_{ti}+{\pi}_{2i}BM{I}_{ti}+{\pi}_{3i} FVInventor{y}_{ti}+{\pi}_{4i} FatInventor{y}_{ti}+{e}_{ti} $$

$$ \begin{array}{c}\hfill \left(L2\ \right)\kern0.5em {\pi}_{0i} = {\beta}_{00}+{\beta}_{01}Ag{e}_i+{\beta}_{02} Gende{r}_i+{\beta}_{03} Educatio{n}_i+{\beta}_{04} Selfefficac{y}_i\hfill \\ {}\hfill +{\beta}_{05} FamilySuppor{t}_i+{\beta}_{06} ChurchSocialSuppor{t}_i+{\beta}_{07} ChurchMessage{s}_i\hfill \\ {}\hfill +{\beta}_{08} ChurchFoo{d}_i+{\beta}_{09} ChurchProgram{s}_i+{\beta}_{010} WorkSocialSuppor{t}_i\hfill \\ {}\hfill +{\beta}_{011} WorkLunc{h}_i+{\beta}_{012} WorkFood{s}_i+{\beta}_{010} WorkProgram{s}_i+{r}_{0i}\hfill \end{array} $$

$$ \begin{array}{c}\hfill {\pi}_{1i} = {\beta}_{10}+{\beta}_{11}Ag{e}_i+{\beta}_{12} Gende{r}_i+{\beta}_{13} Educatio{n}_i+{\beta}_{14} Selfefficac{y}_i+{\beta}_{15} FamilySuppor{t}_i\hfill \\ {}\hfill +{\beta}_{06} ChurchSocialSuppor{t}_i+{\beta}_{17} ChurchMessage{s}_i+{\beta}_{18} ChurchFoo{d}_i\hfill \\ {}\hfill +{\beta}_{19} ChurchProgram{s}_i+{\beta}_{110} WorkSocialSuppor{t}_i+{\beta}_{111} WorkLunc{h}_i+{\beta}_{112} WorkFood{s}_i\hfill \\ {}\hfill +{\beta}_{113} WorkProgram{s}_i+{\beta}_{114} WorkFood sChang{e}_i+{\beta}_{115} WorkProgram sChang{e}_i+{r}_{1i}\hfill \end{array} $$

$$ \begin{array}{c}\hfill {\pi}_{2i}={\beta}_{20}\hfill \\ {}\hfill {\pi}_{3i}={\beta}_{30}\hfill \end{array} $$

All multilevel analyses were conducted using PROC MIXED in SAS 9.3 using restricted maximum likelihood (REML) estimation and excluded all participants with missing data on any of the predictors resulting in 229 participants included in the multilevel models. Significance of variables and variances was assessed at the 0.05 level. Model fit was assessed using deviance scores. Statistical significance for change in model fit was not assessed due to the use of REML. For each model, the reduction in level-1 variance, level-2 intercept variance, and level-2 slope variance were assessed in comparison to the simplest model including the respective random effect as descriptives of the models predictive ability.

## Results

### Demographics

Table 1 reports the demographic characteristics of the participants. The sample consists of 42.9 % men and 57.1 % women. Mean age was 52.2 years (*SD* = 7.78, range 40–70). More than half (53.1 %) of the participants were White and 46.9 % were African American. A large majority (64.6 %) were married. About 40 % of them had no college education; about 26 % completed at least a college degree. At baseline, only 12.9 % of participants were normal or underweight; one third were overweight; more than half (52.7 %) were obese.

### Change over time in individual and environmental factors of fat intake

Change over time was assessed for key individual level variables related to fat intake and those that describe the home, work, or church physical and social environments (Table 2). Fat intake, the main outcome variable, changed significantly from the baseline to the 12 month assessment, with a mean change of 1.53 % (*SD* = 2.53). In addition, BMI changed significantly, showing a small decrease of 0.5 kg/m^2^ (*SD* = 3.10) on average. Self-efficacy for healthy eating remained stable.

**Table 2:** Change in individual and environmental variables (*N* = 245)

	BL		6 months FUP		12 months FUP		
Individual Variables	Mean	SD	Mean	SD	Mean	SD	*p*-value
Fat intake (% of calories from fat)	33.55	2.08	35.59	2.56	35.08	2.51	<0.0001
BMI (kg/m^2^)	31.46	6.59	30.99	6.23	30.96	6.14	0.02
Self-efficacy healthy eating (scale 1–5)	3.65	0.87	3.82	0.71	3.75	0.80	0.07
Home Environment							
Physical							
F&V inventory (out of 7)	3.87	1.61	4.45	1.43	4.02	1.35	0.01
Fat items inventory (out of 3)	2.10	1.15	1.61	1.01	1.65	1.06	<0.0001
Social							
Family support for healthy eating (scale 1–4)	1.89	0.80	1.83	0.82	1.78	0.79	0.07
Work Environment (of those working outside the home)							
Physical							
Lunch facilities (out of 4)	2.80	1.13					
Healthy foods available (yes reported)	*N*	%			*N*	%	*p*-value
- cafeteria	53	32.52			45	28.30	0.86
- vending machine	62	38.27			80	51.28	<0.001
- healthy foods at meetings/events	80	51.28			71	47.65	0.11
Health eating programs (yes reported)							
- nutrition/healthy eating program	41	24.85			51	32.08	0.11
- weight loss program	32	19.51			44	27.67	<0.001
Social	Mean	SD	Mean	SD	Mean	SD	
Social support for healthy eating (scale 1–4)	1.68	0.77	1.89	0.92	1.78	0.87	0.08
Church Environment (of those attending church at least a few times per year)							
Physical							
Healthy eating messages (yes reported)	*N*	%			*N*	%	
- sermon healthy eating	106	49.53			114	55.07	0.33
- church bulletin/newsletter	103	48.58			108	51.43	0.47
Healthy eating programs (yes reported)	42	19.91			43	20.77	0.14
	Mean	SD			Mean	SD	
Healthy foods available (out of 4)	1.75	0.56			1.88	0.55	1.000
Social							
Social support for healthy eating (scale 1–4)	1.40	0.55	1.50	0.68	1.48	0.62	0.07

In the home environment, the household inventories of fruits and vegetables increased significantly over 12 months, with a slight increase from 6 to 12 months. The fat items inventory decreased with a similar pattern, with an average decrease of 0.45 items at the 12 month follow-up. Family social support for healthy eating was not very high (around 1.9 on a 4 point scale) and did not change significantly over time.

In the work environment, changes were significant for the availability of programs to support healthy eating and for one of three indicators of access to healthy food at work. The social support from co-workers was similar to that from family members and did not change significantly within the time frame of this study.

The church environment for healthy eating did not change. There was stability in the number of people reporting healthy eating messages at church (around 50 %) as well as healthy foods available, and healthy eating programs offered (only around 20 %). Social support at church was lower (around 1.5 on a 4 point scale) than for home and work environment and did not change over time.

### Factors of baseline fat intake and change in fat intake over time–Hierarchical Linear Growth Model results

The unconditional growth model (Model 1) estimates the average fat intake at baseline and the average change over time. The results show a significant increase in fat intake over time of 1.25 % per year when not including any variables beyond time.

Model fit improved for each subsequent model. Hence, for reporting results, we will focus on the final most complex model (Model 4). It explained 25.6 % of the variance at baseline. The unexplained variance in the baseline differences was still statistically significant, indicating that there are other factors that can explain differences in fat intake. About 16.7 % of the variance in change over time was accounted for and the unexplained variance was also statistically significant. Furthermore, the model explained 22.8 % over the inter-individual variance; the remaining variance was statistically significant. The residuals for the final model were normally distributed.

The final model estimated the adjusted mean fat intake at baseline was at 35.67 % of calories. Table 3 shows that, on average, holding all other things constant, the fat intake increased by 1.55 % per year due to circumstances not modeled. However, this increase was not statistically significant at the 0.05 level, indicating that change over time is explained by the included time-varying predictors as well as cross-level interactions between individual factors and time. The final growth model gives insight into fat intake cross-sectionally, as well change of fat intake over time.

**Table 3:** HLM Results (*N* = 229)

	Model 1	Model 2	Model 3	Model 4
Fixed effects				
Intercept	33.73***	35.46***	35.46***	35.67***
Level 1-time-varying variables				
Time (in years)	1.25***	1.67***	1.81***	1.55^†^
Individual				
BMI			−0.02	−0.03
Home environment				
F&V inventory			0.12*	0.12^†^
Fat items inventory			0.20*	0.19*
Level 2-time-invariant variables				
Individual				
Age (0 = 40 years)		−0.02	0.0004	−0.002
Gender (0 = male)		−0.55	−0.41	−0.35
Education (0 = high school/less)		0.25	0.35	0.34
Self-efficacy healthy eating			−0.35*	−0.37*
Home environment				
Family support healthy eating			−0.01	−0.01
Church environment				
Church social support			−0.12	−0.11
Church messages			0.64*	0.88*
Church healthy food			−1.18**	−1.13*
Church programs			0.32	0.19
Work environment				
Work social support			0.43	0.20
Work lunch facilities			0.04	−0.24
Work healthy foods			−0.24	0.37
Work programs			−0.06	−0.65
Cross-level Interactions-predicting change in fat intake over time				
Individual				
Time*Age		−0.05**	−0.05**	−0.05**
Time*Gender		0.70*	0.67*	0.62*
Time*Education		−0.38	−0.46	−0.50
Time*Self-efficacy healthy eating				0.06
Home environment				
Time*Family social support				0.02
Church environment				
Time*Church social support				−0.01
Time*Church messages				−0.46
Time*Church healthy food				−0.05
Time*Church programs				0.36
Work environment				
Time*Work social support				0.35
Time*Work lunch facilities				0.40
Time*Work healthy foods				−0.90*
Time*Work programs				−0.86*
Time*Work healthy foods change				0.63^†^
Time*Work programs change				−0.24
Random effects				
τ_00 (intercept)_	2.09***	2.13***	1.67***	1.68***
τ_11 (Time)_	0.78*	0.59*	0.74*	0.65*
σ^2^	3.28***	3.25***	3.19***	3.19***
Model fit				
Reduction in τ_00_	7.4 %	5.7 %	26.0 %	25.6 %
Reduction in τ_11_		24.4 %	5.1 %	16.7 %
Reduction in σ^2^	20.6 %	21.3 %	22.8 %	22.8 %
Deviance	3170.8	3165.0	2974.0	2947.3
AIC	3176.8	3171.1	2980.0	2953.3
BIC	3187.3	3181.5	2990.3	2963.6

Note: All models account for clustering of participants in counties. † *p* < 0.10 * *p* < 0.05 ** *p* < 0.01 *** *p* < 0.0001

#### Factors of baseline fat intake

There were several individual variables of baseline fat intake modeled. However, only self-efficacy for healthy eating was significantly related to fat intake, with higher self-efficacy resulting in lower fat intake (β = −0.37, SE = 0.17). Age, gender, and education did not explain differences of fat intake at baseline.

In the home environment, the fat items inventory was a significant factor in explaining fat intake. While the number of unhealthy high-fat items decreased over time (Table 2), having a larger variety of unhealthy high-fat items in the home was related to higher fat intake and vice versa (β = 0.19, SE = 0.09). Neither the fruit and vegetable inventory nor family support for healthy eating is predictive of fat intake.

Some of the church environment variables predicted fat intake. Participants who were exposed to messages about healthy eating at church had a higher fat intake than those who did not hear such messages at church or did not attend church (β = 0.87, SE = 0.34). Those reporting better access to healthy food at church had a significantly lower fat intake (β = −1.13, SE = 0.47).

Neither physical nor social components of the work environment had a significant effect on the average fat intake.

#### Factors of change in fat intake

The final model also shows which variables have an impact on change in fat intake. When looking at individual variables, age (β = −0.05, SE = 0.02) and gender (β = 0.62, SE = 0.30) have a significant effect on the change in fat intake over time. Older participants increased their fat intake over time, but less so than younger ones. In addition, women increased their fat intake more than men, by 0.62 % over 12 months. Education and self-efficacy for healthy eating did not predict change in fat intake.

In the home environment, the fat item inventory (as mentioned earlier) had an impact on fat intake (β = 0.19, SE = 0.09) with having more unhealthy items in the home predicting higher fat intake. No other home environment factors were significantly influencing change in fat intake.

In the work environment, there was a positive influence of having healthy foods available (β = −0.90, SE = 0.38) on the change in fat intake. Participants who had access to healthy foods at work had a lower increase in fat intake (by 0.90 % points over 12 months) than those who did not. In addition, participants who had access to work programs regarding healthy eating had a lower increase in fat intake (β = −0.86, SE = 0.41). Participants who had both access to healthy foods at work and work programs for healthy eating decreased their fat intake over time.

None of the variables from the church environment explained change in fat intake.

#### Results at a glance

Figure 2 shows all factors investigated in this study. The diagram shows which environments and also which variables within each environment changed over time. Significant determinants of fat intake and change in fat intake are noted as well.

## Discussion

This longitudinal observational study offers insight into determinants of fat intake in general as well as change in fat intake in a rural population in the Southeast region of the US. Overall, the participants increased their fat intake over the 12 month period of the study. Several interesting observations can be made when looking at the change in individual factors as well as different environmental factors of fat intake over the period of 1 year. Individually, there was an average decrease in BMI from self-report. However, the physical environments of the home (inventories of fruits and vegetables as well as unhealthy snacks) changed significantly over the 12 month study period, which might have increased the intake of low-energy dense foods. The church environments were stable both in physical and social factors related to fat intake. There were some notable changes in the work environments: an increase in healthy foods in vending machines and weight loss programs at work sites.

The multilevel model allows us to draw conclusions on how these factors interact and help explain fat intake in general as well as change in fat intake over time.

When looking at fat intake at baseline, we found an impact of the home and the church environment, but not the work environment. A recent research study conducted in Australia suggests that food environments near work places might influence eating behaviors [53]. It would be meaningful to investigate impact of the food environment at the work facility in conjunction with access to food in the workplace neighborhood on eating behaviors, distinguishing between food prepared away from home from food prepared at home and taken to work due to significant nutritional disadvantages of food prepared away from home [54].

Not surprisingly, we found having fatty items in the home is related to higher fat intake. This means that changing the home environment by reducing the number (and amount) of high-fat items has a positive impact on the eating behavior resulting in lower fat intake. This finding confirms similar findings from many other studies [4, 55].

In addition, higher self-efficacy for healthy eating is associated with lower fat intake. Social support in all three settings was relatively modest and we did not see an impact for family, church or workplace social support on fat intake. While interventions should include components aimed at increasing self-efficacy for healthy eating, it would be also of interest to see if collective social support [56] might have an impact on fat intake rather than family social support. Our lack of social support findings may also be related to measurement as our church and workplace social support measures were shortened considerably from the original scales. Interestingly, while prior research has generally shown an effect of social support on healthy eating, another one of our studies in the same region found no association between family social support for healthy eating and nutrition behavior [27, 35, 57].

While we did not see an impact of the work environment on fat intake, having healthy food available at church was associated with lower fat intake. Several evidence based programs to improve the church eating environment are available [58, 59].

Because there was a significant change in fat intake during this 1 year study, it allows a look at what individual and environmental factors predict change in fat intake. While the overall trend showed people increasing their fat intake, there were several time-invariant factors that were associated with a decrease in fat intake over time from three areas: individual factors, as well as the work environment. When looking at individual factors of change in fat intake, we found that older people decreased their fat intake more than younger people and women increased their fat intake more than men. Positive physical eating environments at work (offering healthy foods and programs for healthy eating) also had positive impact on change in fat intake. Neither the home nor the church environments were predictive of the change in fat intake.

The current analysis also indicates that there are additional variables of fat intake in general and change in fat intake over time that we have not measured. Collecting more in-depth dietary intake data, for example through 24 dietary recalls would allow to investigate the relationships between fat intake, fruit and vegetable intake, and BMI more in-depth. Future research should investigate additional variables on the individual level as well as from the three environments discussed here. In addition, other environments such as homes of family members and friends might play a significant role in fat intake. Interventions might aim at improving the physical environments and investigate the impact on healthy eating. Churches seem to provide very stable environments and change could reach many people.

In addition, this study shows stability and variability in the physical and social home, church, and work environments related to eating behaviors.

The lack of change in many of the environmental variables such as social support environments is of interest. Improving the social support for healthy eating in one or several of these environments might have an impact on fat intake, but more research is needed. In addition, there was no change in the physical church environment reported. As mentioned earlier, there are evidence based programs for improving the healthy eating environments in churches available focusing on fruit and vegetable intake [58, 60, 61]. Future research should investigate long-term impact of such changes on fat intake.

The changes observed in the work environment (i.e. availability of healthier food options and weight loss programs) are promising as is their impact on change in fat intake. Strengthening these efforts and widening their use might be a viable avenue for reducing fat intake. It would also be of interest to look the food environment in work place neighborhoods.

This study has several limitations. Sampling was not random and locations chosen to recruit participants might have resulted in a biased sample. In addition, the longitudinal study only included high-risk participants. Hence, the results are neither generalizable to the general population in rural southwest Georgia nor other areas in the United States. The self-report nature of data collection might have introduced reporting biases, including social desirability bias. Measures of variables were brief, in order to be acceptable to respondents. Furthermore, there are many other variables, both individual and in the three key environments, that could have been measured and enhanced the understanding of the determinants of fat intake and change thereof.

## Conclusion

Despite its limitations, this study contributes important knowledge about home, work, and church environments’ impact on fat intake. Very few studies have looked at the interplay of influences from all three environments over time, especially for an at-risk population. Future studies in different populations are needed to investigate the combined influence of multiple key environments on nutrition behaviors.
